# Dr. Black’s Review

**Published:** 1898-08

**Authors:** 


### Editorial.

### DR. BLACK'S REVIEW.

In the June number of the *Dental Cosmos* Dr. Black reviews his reviewers as they appeared in the December, 1897, number of the International Dental Journal. That this will be regarded as a satisfactory reply seems doubtful, for his words, while respectfully phrased, have a sarcastic flavor little in keeping with scientific writing, and not at all favorable to the establishment of a scientific basis worthy the acceptance of the entire dental profession.

Dr. Black was fully aware that his deductions from his experiments would arouse antagonism everywhere, for these were in direct opposition to the clinical experience in dentistry for many decades. It was therefore proper that men of large experience should give their views, and that these should be carefully considered.

It is not by any means the first time that laboratory and clinical experience have come into direct antagonism. It is possible that both may be right, and it is therefore unsafe for either side to assume too much. The former demands that all experience shall bow to its dictum, and that no truth is possible except it be demonstrated by actual experiment. That this is an untenable position needs no argument. It is impossible to spare either method, and he who attempts it will find himself very speedily in a maze of difficulties.

Dr. Black pays special attention to the essay of the editor of this journal, and says, "Dr. Truman thinks I may have reported truly on the teeth in hand, but that the right teeth were not obtained, for, he says, page 797, 'If, on the other hand, teeth had been selected from a mouth recognized as having chalky teeth, and these be compared with the aforementioned, and the result had been the same, it would not have been possible to have offered any criticisms, for even personal observations must give way to the crucible of analysis.'

"No other person, to my knowledge, has resorted to such an argument as this. It amounts to a simple denial that I did the thing that I took especial pains to do and so expressed. . . . On page 358, *Dental Cosmos*, May, 1895, under the head of 'Collection of

Material,' it will be found that capable dentists who assisted me, and who are named on page 366, were instructed to give the 'nativity, age, state of the general health, to *class the teeth as bad, fair, or good; as to occurrence and rapidity of caries*, record the condition of gums, etc.,' thus fulfilling to the letter the requirements Dr. Truman would impose and which he sees fit to ignore."

This does not meet the question and is an unfair statement of facts, for Dr. Truman has invariably quoted this paragraph in the several articles prepared by him upon this subject. The construction which he places upon this differs from that of Dr. Black. It is assumed by him that this is the weak point in Dr. Black's experiments, and had this link in the chain been perfect there would have been no cause for criticism as to methods. It is repeated, therefore, that this plan of collecting material was a weak starting-point. *The teeth that the dental profession calls soft and chalky are not to be had for the asking, and it must be exceedingly rare that they come into the dentist's hand through extraction*, as they are mainly to be found from the ages of twelve to twenty. This it is thought will be universally conceded. Dr. Black cannot expect that intelligent practitioners will accept the teeth sent to him as representing their idea of soft, chalky teeth. The arrangement as outlined by him was doubtless the best he could do, but to base a complicated series of experiments upon a collection thus made seems to Dr. Truman to be placing in jeopardy a scientific reputation worthily earned and universally honored.

Dr. Truman is again taken to task for inconsistency, for Dr. Black says, "Dr. Truman denies his own statement, seemingly, when he says, page 798, 'Caries is not confined to teeth of poor structure; indeed, it may be said, with some degree of truth, that this is no indication of the character or even the extent of the destruction.' I submit the doctor should guard his statements better than this." Exactly what Dr. Black sees in this sentence inconsistent with anything previously stated will remain a mystery to Dr. Truman. The dentist who does not know that all teeth, given the "opportunity," as Dr. Black states it, will decay, must have lost the faculty of observation, if it ever existed. That all teeth are liable to decay is an axiomatic phrase, but the capability of distinguishing the conditions that existed prior to caries from examinations out of the mouth is an unknown quantity.

Further, he states that "Dr. Truman then runs off in a wild chase after leptothrix buccalis in an effort to controvert jointly Dr. Williams's paper and my own." Exactly what is meant by a "wild chase" is not apparent. It may mean, in Dr. Black's estimation, a chase after something non-existing. The point will not be contested. If, however, he means to convey the idea that Dr. Truman does not know much of the subject, he may be kindly informed that that individual has devoted many years of his life to histological work, and deems himself quite capable of at least forming an intelligent opinion upon any subject relating thereto. The question cannot be dismissed by a wave of the hand, the dash of a pen, or sarcastic allusions, but must be met. Unfortunately for Dr. Black, and indirectly for Dr. Williams, the following admission of the former concedes all that Dr. Truman claimed: "They [leptothrix] have therefore long ago been dropped out of consideration as caries-producing fungi. It is fungi of a different sort that are the active agent in the production of caries." Dr. Truman's contention could not have been expressed more plainly. Dr. Williams's original pictures and his slides, since seen by the writer, demonstrated that the "gelatinous microbic plaques," as Dr. Black calls them, were plentifully distributed, and that they were leptothrix threads *and not the micro-organisms of caries*, hence not acid-producing. Dr. Black having acknowledged this, and Dr. Williams, in his apple story, brought to bear on Dr. Truman, having equally acknowledged that the evidence was altogether circumstantial, the entire question may be left where it properly belongs,—to future examination.

Dr. Black further says, "When Dr. Williams showed the presence of these organisms in the gelatinous microbic plaques upon the teeth, together with the effects upon the enamel beneath, it was not incumbent upon him or any one else to prove again that these were acid-producing organizations, and that they furnished the acid which caused the chemical disintegration which was apparent." This might be all true provided Dr. Williams did show the acid-producing organisms, but Dr. Truman failed to discover these in any appreciable numbers, but did find leptothrix buccalis profusely developed, and these threads, he may repeat, have not been identified as acid-producers.

If there is any statement in this review of Dr. Black more worthy of astonishment than another it is this: "It seems to me too late for any one to argue that caries of the teeth is caused by acids freely dissolved in the saliva." The school of Dr. Black and Dr. Williams, in which Dr. Truman holds much in common, has declared over and over again that environments, and not density, have much to do with the production of caries, or, to place it more correctly, the acid conditions generated in the oral secretions act chemically upon the teeth and invite the formation of caries-producing organisms. It is possible that Dr. Truman fails to understand Dr. Black, but, as comprehended, the statement is inconsistent with much heretofore given, as it is equally untrue in fact.

There is no desire to extend this controversy further, but it is imagined the dental profession will not be converted to Dr. Black's deductions, or to Dr. Williams's peculiar polemical methods, as long as they confine their work to the laboratory and hold as practically valueless all clinical observations. From these leaders in laboratory work we have a right to demand facts and not thoughts. Deductions properly made must always have a value, but a story of an apple thief in Dr. Truman's apocryphal garden may be amusing and entertaining to professional groundlings, but can have no value as a means of advancement of our knowledge in the science of our profession.

